# Temporary clamping of drain combined with tranexamic acid reduce blood loss after total knee arthroplasty: a prospective randomized controlled trial

**DOI:** 10.1186/1471-2474-13-124

**Published:** 2012-07-20

**Authors:** Keerati Chareancholvanich, Pichet Siriwattanasakul, Rapeepat Narkbunnam, Chaturong Pornrattanamaneewong

**Affiliations:** 1 Department of Orthopedic Surgery, Faculty of Medicine, Siriraj Hospital, Mahidol University, Bangkok, Thailand

**Keywords:** Total knee arthroplasty, Bleeding, Drain, Clamp, Tranexamic acid

## Abstract

**Background:**

Total knee arthroplasty (TKA) is associated with a significant blood loss. Several methods have been reported to reduce postoperative blood loss and avoid homologous blood transfusions. In this study, we investigated the efficacy of temporary clamping of the drain either or not in combination with tranexamic acid administration for controlling blood loss after TKA.

**Methods:**

The prospective, randomized, and double-blinded study was conducted in our institute. Total of 240 patients, who diagnosed primary osteoarthritis and scheduled to undergo a primary TKA,,were randomized into one of the four groups: Group A or control group, the drain was not clamped and the patient received a placebo; Group B, the drain was not clamped and the patient received tranexamic acid; Group C, the drain was clamped and the patient received a placebo; and Group D, the drain was clamped and the patient received tranexamic acid. The volume of drained blood at 48 hours postoperatively, the decreasing of hemoglobin (Hb) level at 12 hours postoperatively and the number of patients requiring blood transfusion were recorded and compared.

**Results:**

The mean postoperative volumes of drained blood and the amount of blood transfusion in the three study groups (group B, C and D) were significantly lower than those in the control group (p < 0.05), which group D had the lowest values. Furthermore, group B and D could maintain the Hb level better than group A and C (p < 0.001). In terms of blood transfusions rate, although the patients in group D required transfusion less than group A and C (p < 0.05), there was no significant difference between group D and B. The relative risks for transfusion requirement were 4.4 for group A, 1.4 for group B and 3.0 for group C when compared to group D.

**Conclusions:**

The clamping of drain combined with tranexamic acid administration could reduce postoperative blood loss and blood transfusion after TKA, significantly greater than using tranexamic acid or drain clamping alone.

**Trial registration:**

ClinicalTrials.gov NCT01449552

## Background

Total knee arthroplasty (TKA) is one of the effective treatments for severe osteoarthritic knee. The goals of this procedure include correcting deformity, restoring pain-free motion and maintaining stability of the knee. Currently, TKA is widely acknowledged to be one of the most successful and cost-effective procedures in orthopedic practice. However, considerable blood loss after TKA is still problematic and postoperative blood transfusion carries a substantial risk of both immunologic reaction and transmission of diseases [1]. Blood transfusion also involves an additional cost, so a reduction in its use is very important.

Several methods have been reported to reduce postoperative blood loss and avoid homologous blood transfusion. The methods shown to be effective include autologous blood transfusion [2], postoperative blood salvage [3-5], intramedullary femoral plug [6], hypotensive anesthesia [7], cryotherapy with Robert Jones bandage [8], fibrin tissue adhesive [9,10], drain clamping [11-18], and tranexamic acid administration [19-29].

Although most of the surgeons performed TKAs with the use of tourniquet, postoperative blood loss still occurred. Most of the blood loss in TKA occurs during the first few postoperative hours [30]. The drain clamping is one of the methods proposed to reduce blood loss in early postoperative period of TKA. Various protocols of this method have been reported [11-18]. For examples, Sakihara et al. [11] infused 50 ml of saline containing antibiotic into the knee joint through the drain tube and clamped the drain for 1 hour. Shen et al. [15] clamped the drain for 4 hours. Tsumara et al. [16] clamped the drain for 30 minutes with intra-articular injection of saline with adrenaline. Prasad et al. [17] recommended using 2-hour clamping of the drain and release for 10 minutes. All of these previous studies proved that the uses of their methods could reduce blood loss and transfusion requirement following TKA. Nevertheless, some studies reported that drain clamping had no benefit in routine TKA [31].

Tranexamic acid is a synthetic anti-fibrinolytic drug used to prevent bleeding. Initially, fibrinolysis is stimulated by surgical trauma [32,33] and further augmented by the use of a tourniquet [34,35]. This phenomenon may lead to increase blood loss after TKA, especially during the first few postoperative hours. Administration of tranexamic acid can inhibit the activation of plasminogen to plasmin [36,37] by blocking the lysine binding sites of plasminogen to fibrin, which results in inhibition of fibrinolysis [38,39].

Several studies reported that tranexamic acid could reduce either blood loss or blood transfusions [19-23]. While some studies demonstrated its efficacy in reducing blood loss but not reducing blood transfusion [24,25]. Hynes et al. [26] found that tranexamic acid also could reduce the decreasing of hemoglobin (Hb) level following TKA. Nevertheless, Engel et al. [40] revealed that tranexamic acid did not cause a significant modulation of fibrinolysis variables or a significant reduction of postoperative bleeding and transfusion requirements.

Based on the level I evidences, meta-analyses showed that intravenous tranexamic acid appears effective and safe in reducing allogeneic blood transfusion and blood loss in TKA without increasing the risk of thromboembolic complications [28,29]. To our knowledge, no study has compared the efficacy of drain clamping combined with tranexamic acid administration in the control of bleeding following TKA. Thus, the purpose of this study was to evaluate the efficacy of drain clamping alone, tranexamic acid alone and the combination of these two modalities in the control of bleeding following TKA.

## Methods

This study was designed as a prospective, randomized, double-blinded, controlled trial and approved by the Ethics committee of Siriraj Hospital. All patients gave their written informed consent for participation in the study. Between June and November 2008, 240 patients who scheduled to undergo a unilateral primary TKA were enrolled in the study. The patients aged less than 85 years and diagnosed primary osteoarthritic knees were included. The exclusion criteria were the patients who had secondary osteoarthritis (such as rheumatoid arthritis, post-traumatic arthritis, gouty arthritis, post-septic arthritis), high risk medical co-morbidity, history of thromboembolic disease, bleeding disorder, known allergy to tranexamic acid, and receiving the anti-coagulant drugs.

A computer-generated list was created by the blocks-of-12 randomization method. The patients were randomly assigned to one of the four groups: group A was non-clamping of drain and placebo administration, group B was non-clamping of drain and tranexamic acid administration, group C was clamping of drain and placebo administration and group D was clamping of drain and tranexamic acid administration. The concealed envelopes were used to blind the surgeon and participants for the randomization sequences and opened by a second year resident, who was not involved in the study.

Our senior author (KC), who is experienced in TKA, performed all of the operations on patients who were under spinal anesthesia. A pneumatic tourniquet with a pressure of 350 mmHg was inflated after limb exsanguinations. A straight skin incision and a mini-medial parapatellar capsular incision were used in all knees. The patellas were not resurfaced. A Nexgen LPS-Flex fixed-bearing design (Zimmer, Warsaw, Indiana), inserted with cement, was used. An intramedullary femoral alignment rod was used in all cases, and the femoral canal was filled with a bone plug before the prosthetic implantation.

In group B and D that received tranexamic acid protocol, 10 mg/kg of intravenous tranexamic acid (Transamin; 250 mg/5 ml, OLIC, Thailand) was administrated at 10 minutes before inflating the tourniquet and 10 mg/kg at 3 hours postoperatively. Then, 1500 mg per day of oral-formed tranexamic acid (Transamin; 250 mg/capsule, OLIC, Thailand) was given for 5 days after operation. While in group A and C, the placebo (equivalent volume of physiologic saline combined with a starch capsule) was administrated instead.

At the end of the operation, a number-10-gauge drain was placed intra-articularly and was connected to the Ultravak pressure drainage bottle (Poly Medicure Limited, India). After wound closure, a compressive Robert Jones bandage and a posterior splint were applied. Following the release of tourniquet pressure, the drain in group A and B was immediately released and the drain in group C and D was clamped following our protocol, so called 3-hour-interval clamping technique (clamped for 3 hours, released for 3 hours, reclamped for 3 hours and then the clamp was run continuously).

The patient was encouraged to perform a mechanical ankle pumping exercise to prevent deep vein thrombosis as soon as possible after surgery. The bandage, splint and Foley catheter were removed on the first post- operative day. On the same day, the range-of-motion exercise, an isometric/isotonic quadriceps exercise, a straight leg rising exercise and a walking exercise were initiated under the control of a physiotherapist.

The amount of drained blood was recorded at 48 hrs. All suction drains were removed 48 hours postoperatively. The Hb levels were determined preoperatively and 12 hours postoperatively. The patients received a transfusion of one unit of packed red cells (PRC), if their Hb levels decreased to <10 g/dL or if the compromised clinical criteria (e.g., tachycardia, hypotension, or symptoms of anemia that were relative to the preoperative medical condition of the patient) necessitated transfusion. If Hb levels decreased to <8 g/dL, the patient received two units of PRC. Following the blood transfusions, we reevaluated Hb levels at 6 hours after the end of the transfusion period, and blood replacement was considered again using the same criteria as outlined above. At 48 hours after the operation, the Hb levels of all patients were recorded. Clinical thromboembolic events and wound complications were also examined. All of the patients were discharged from the hospital on the fifth day after the operation.

### Statistical analysis

The data were analyzed using the commercially available SPSS statistics software, version 13.0. Quantitative data were presented as the mean ± standard deviation and differences in the means among the four groups were analyzed using an analysis of variance (ANOVA). We determined differences in sex and the number of patients requiring blood transfusions using the Chi-square test. The risks of blood transfusion compared between groups were analyzed using logistic regression. P values < 0.05 were regarded as statistically significant.

## Results

Over a six-month period, 240 patients (60 patients per group) were available for analysis. The mean age of the patients was 69.8 ± 6.8 (range from 53–84) years. The preoperative data included age, gender and preoperative Hb were comparable among the four groups (Table 1). The mean postoperative volumes of drained blood were 1182 ± 411 ml in group A, 724 ± 246 ml in group B, 821 ± 337 ml in group C, and 526 ± 222 ml in group D. Group A had significantly higher volume of drained blood than the others (p < 0.001) but there were no significant differences in those between group B and C (p = 0.37). The lowest drained-blood volume was found in group D (p < 0.001) (Table 2).

**Table 1:** Pre-operative data

**Characteristics**	**Group A**	**Group B**	**Group C**	**Group D**	**p-value**
	(n = 60)	(n = 60)	(n = 60)	(n = 60)	
Male : Female	8 : 52	9 : 51	10 : 50	8 : 52	0.946
Age (yr)^*^	69.8 ± 6.3	69.4 ± 6.3	68.9 ± 7.5	70.1 ± 7.2	0.418
Pre-op Hb (g/dl)^*^	12.5 ± 1.1	12.4 ± 1.1	12.4 ± 1.1	12.6 ± 1.0	0.515

* Data was presented as mean ± standard deviation.

Pre-op Hb = Preoperative hemoglobin.

**Table 2:** Blood loss and transfusion requirement

**Values**	**Group A**	**Group B**	**Group C**	**Group D**	**p-value**
	(n = 60)	(n = 60)	(n = 60)	(n = 60)	
Volume of drained blood	1182 ± 411	724 ± 246	821 ± 337	526 ± 222	< 0.001^a^
(ml)^*^	(420–2260)	(230–1330)	(230–2020)	(180–960)	
Decreasing Hb at 12 hours	3.3 ± 0.9	2.1 ± 0.6	2.8 ± 0.8	1.8 ± 0.7	< 0.05^b^
(g/dl)^*^	(1.3-5.3)	(0.9-3.8)	(0.9-4.4)	(0.4-3.6)	
Number of patients requiring blood transfusion	53 (88.3%)	34 (56.7%)	49 (81.7%)	23 (38.3%)	< 0.05^c^
PRC transfusion (unit)^*^	1.8 ± 1.0	0.7 ± 0.7	1.3 ± 0.9	0.4 ± 0.5	< 0.05^d^
	(0–4)	(0–2)	(0–3)	(0–2)	

* Data was presented as mean ± standard deviation and range.

^a^ Statistical difference among all groups (except between group B and C, p = 0.37).

^b^ Statistical difference among all groups (except between group B and D, p = 0.07).

^c^ Statistical difference among all groups (except between group A and C, B and D).

^d^ Statistical difference among all groups (except between group A and C, p = 0.05).

Hb = hemoglobin, PRC = packed red cell.

The mean levels of decreasing Hb at postoperative 12 hours were 3.3 ± 0.9, 2.1 ± 0.6, 2.8 ± 0.8 and 1.8 ± 0.7 g/dl in group A, B, C and D, respectively. Group B, C and D had significantly lower levels of decreasing Hb than those in group A (p < 0.05). Group B and D could maintain the Hb level better than group C (p < 0.001), however; there was no significant difference between group D and B (p = 0.07) (Table 2).

The amount of PRC transfusion units required in group B and D were significantly lower than group A and C (p < 0.001). There was no significant difference between group A and C (p = 0.05). The patients who received the least blood transfusion (0.4 ± 0.5 unit) were in group D (significantly lower than group B, p < 0.04) (Table 2). In the number of patients requiring blood transfusion, group D had significantly lower number than group A and C (p < 0.05). However there were no significant difference between group D and B (Table 2). The relative risks for transfusion requirement were 4.4 for group A, 1.4 for group B and 3.0 for group C when compared to group D.

In terms of complications, no wound infections or clinical venous thromboembolisms were detected in all groups. Although three patients (each one from group A, B and D) developed postoperative ecchymosis around the knees, this resolved spontaneously.

## Discussion

The significant blood loss and risk for blood transfusion are important features that must be considered in TKA. Complications after allogeneic blood transfusions have been well reported in the previous literature [1]. Several methods have been reported to reduce blood loss and blood transfusion after TKA [2-29]. However, the best method remains unknown. Either drain clamping [11-18] or tranexamic acid administration [19-29] is the simple method that we interested. The combined effect of these two methods was still unknown. We therefore aimed to study the efficacy of drain clamping alone, tranexamic acid alone and the combination of these two modalities in the control of bleeding following TKA.

Because most of the blood loss in TKA occurs during the first postoperative day (71.1 and 84% in the first 6 and 12 hour after operation, respectively) [41,42], it seems reasonable to clamp the drain in the early postoperative period to create a tamponade effect and to control blood loss. Although various protocols for drain clamping have been reported in the literature [11-18], we have established a new interval clamping protocol for reducing blood loss in TKA. Kiely et al. [31] concluded that 2 hours of drain clamping has no benefit in routine TKA. Thus, a longer period of drain clamping may be required. However, hematoma and wound complications must be taken into consideration for long-period clamping protocols. To balance between creating a tamponade effect and reducing wound complications, an interval clamping protocol using a 3-hour interval pattern that routinely used in our institute was selected for this study. From the results of this study, our drain clamping technique alone could reduce more blood loss and keep higher 12-hour Hb level than the non-clamping group. Nevertheless, the use of this protocol did not affect the reduction of transfusion requirement, either amount or rate.

Fron non-pharmacological to pharmacological method, there are four routes for administrating tranexamic acid in order to reduce blood loss in TKA: oral, intramuscular, intravenous, and intra-articular [27]. The time taken for maximum plasma levels of tranexamic acid to be reached has been reported to be 2 hours for oral, 30 minutes for intramuscular and 5–15 minutes for intravenous administration [43,44]. Many clinical studies reported tranexamic acid reduced blood loss or transfusion requirements when given on deflation of the tourniquet with a repeated dose postoperatively [19,20,22-29,45]. Tanaka et al. [27] concluded that the hemostatic effect was best when tranexamic acid was given once 10 minutes before surgery and once upon deflation of the tourniquet. The administration before the operation gave more hemostatic effect than administration upon deflation of the tourniquet. Pharmacokinetic studies [43,44,46,47] indicated that a dose of 20 mg/kg of tranexamic acid is suitable for TKA since therapeutic levels could be maintained for approximately 8 hours after the operation, which covered the period of hyperfibrinolysis in cases of increased blood loss. Thus, we also used a dose of 20 mg/kg of intravenous tranexamic acid that divided into two parts: 10 mg/kg at 10 minutes before tourniquet inflation and another 10 mg/kg at 3 hours postoperatively. Then, an oral form of tranexamic acid was given for 5 days in order to control bleeding during rehabilitative training.

With using our tranexamic acid regimen, we found that it significantly reduced about 40% of blood loss, compared to those in control group. This result was in the same way of previous studies that reported on 30-50% of blood loss reduction [19-24,27]. Furthermore, our protocol also showed the superior efficacy in reducing amount and rate of blood transfusion, and maintaining the Hb level, over the control group.

The hemostatic effects of the tranexamic acid alone were significantly better than the drain clamping alone with regards to the level of decreasing Hb, amount of blood transfusion and number of patients requiring blood transfusion, despite the volume of drained blood between these two methods was not significantly different. These findings confirmed that some blood might remain around the knee joint, leak through the wound, or diffuse into the soft tissue, especially when the drain was clamped [13,48].

Importantly, this study also verified the efficacy of the administration of tranexamic acid combined with the drain clamping, which had never been studied before. Compared to the control group, the use of this combination could reduce the volume of drained blood up to 55% that significantly more than using the tranexamic acid alone (40% of reducing blood loss) or the drain clamping alone (30% of reducing blood loss). Furthermore, this combined method provided the lowest amount of transfusion unit. For the efficacy in maintaining the Hb level and reducing the rate of transfusion requirement, the use of our tranxenamic acid protocol combined with the 3-hour interval drain clamping was proved to give more benefit than using drain clamping alone. Nevertheless, it was not significantly different from using tranexamic acid alone. The superior effect of tranexamic acid over clamping of the drain might explain this phenomenon. After calculating the relative risks, the number of patients required blood transfusion in control group was 4.4-fold, in drain clamping alone was 3.0-fold and in tranexamic acid alone was 1.4-fold when compared to the combined method.

There are some limitations in this study. First, the female to male ratio was high because most patients undergoing TKA in our country are females. Female patients may have less preoperative hemoglobin than male, which may affect the rate of blood transfusion. Nevertheless, after randomization, the female-to-male ratios and preoperative hemoglobin levels were not different among the four groups. Second, we used only clinical evaluations to evaluate the thromboembolic complications that could not detect asymptomatic deep vein thrombosis and pulmonary embolism. The correlation of using tranexamic acid and venous thromboembolism remains unknown. Third, although we proposed a 5-day regimen of oral formed tranexamic acid might assist to control blood loss during rehabilitation, there were no measurement tools to assess this hypothesis. Finally, we focused only on the efficacy in controlling blood loss. This report did not include functional scoring systems or patient satisfaction.

## Conclusion

This study proved that 3-hour-interval drain clamping technique significantly reduced postoperative blood loss and our tranexamic acid administration protocol significantly reduced blood loss and blood transfusion after TKA. The use of these two combined methods demonstrated the better hemostatic effect than using tranexamic acid alone or drain clamping alone.

## Competing interests

The authors declare that they have no competing interests.

## Authors’ contributions

KC made substantial contributions to conception and design, data acquisition, data analysis, and data interpretation. PS made substantial contributions to conception and design, data acquisition, data analysis, data interpretation and drafting the manuscript. RN and CP made data analysis, data interpretation, drafting the manuscript and revising it critically for important intellectual content. All authors read and approved the final manuscript.

## Authors’information

Associate Prof. Keerati Chareancholvanich, MD.; Pichet Siriwattanasakul, MD.; Rapeepat Narkbunnam, MD. and Chaturong Pornrattanamaneewong, MD. are the instructors at Department of Orthopedic Surgery, Faculty of Medicine, Siriraj Hospital, Mahidol University, Bangkok, Thailand.

## Pre-publication history

The pre-publication history for this paper can be accessed here:

http://www.biomedcentral.com/1471-2474/13/124/prepub
